# Completely endophytic renal tumor: A laparoscopic approach

**DOI:** 10.1590/S1677-5538.IBJU.2017.0534

**Published:** 2018

**Authors:** Victor Espinheira Santos, Rafael Ribeiro Meduna, Wilson Bachega, Gustavo Cardoso Guimarães

**Affiliations:** 1 Serviço de Urologia, Departamento de Cirurgia Pélvica, AC Camargo Cancer Center, São Paulo, SP, Brasil

## Abstract

Kidney cancer is the third most common urologic malignancy and a 2% annual increase in the incidence has occurred over the past two decades, largely because of the increased utilization of imaging. The majority of these tumors are small, so the indications for nephron-sparing surgery and for minimally invasive surgery are continually expanding. Complex kidney lesions, such as those completely endophytic, are still a challenge even for experienced surgeons.

Our objective is to demonstrate the operative technique for laparoscopic partial nephrectomy with the aid of intra-operative ultrasound in a man with a totally endophytic renal lesion.

Case: A 52 years old man, asymptomatic, with incidental renal mass of 2.9 cm, completely endophytic (R.E.N.A.L score 9p) submitted to partial laparoscopic nephrectomy. Surgical time was 2 hours, with 20 minutes of ischemia. Pathological anatomy confirmed tumor of clear cells T1a, free margins.

## ARTICLE INFO

**Available at: http://www.intbrazjurol.com.br/video-section/20170534_Santos_et_al**

**Int Braz J Urol. 2018; 44 (Video #16): 1050-1050**

